# Molecular Imaging in Nanomedical Research 2.0

**DOI:** 10.3390/ijms232113011

**Published:** 2022-10-27

**Authors:** Manuela Malatesta

**Affiliations:** Department of Neurosciences, Biomedicine and Movement Sciences, University of Verona, 37134 Verona, Italy; manuela.malatesta@univr.it

Over the last two decades, imaging techniques have become irreplaceable tools in nanotechnology: electron microscopy techniques are routinely used to observe the structural features of newly manufactured nanoconstructs, while light and electron microscopy, magnetic resonance imaging, optical imaging, positron emission tomography, and ultrasound imaging allow dynamic monitoring of the biodistribution, targeting and clearance of nanoparticulates in living systems, either for the whole organism or at the level of single cells, tissues and organs [1].

The first Special Issue, “Molecular Imaging in Nanomedical Research”, contained five research articles and two reviews on diverse imaging techniques applied to the development of novel nanoconstruct-based strategies for diagnosis and therapy [2]. In this Special Issue, the collection continues with three research articles and six reviews that update and enrich our knowledge of the basics and applications of molecular imaging in nanomedical research.

Chang and co-workers [3] used polyamine-based PEG-polyplex nanomicelle as a delivery system for Runx1 mRNA, aiming to alleviate the spinal disc hydration loss in a rat disc degeneration model. The authors examined the endocytosis of mRNA-loaded polyplex nanomicelles in vitro, and performed in vivo evaluation of the mRNA delivery efficacy and expression persistence, the disc shrinkage, and the hydration of the disc extracellular matrix. The administration of Runx1 mRNA via polyplex nanomicelles significantly improved the hydration content, the disc space and the production of extracellular matrix, suggesting that this procedure may be a promising strategy in regenerative medicine.

Su and co-workers [4] aimed to develop an efficient near-infrared imaging method to monitor the kinetics of single-domain antibodies from camelids (VHH) and VHH-conjugated iron oxide nanoparticles in mice. A hybrid approach was used, in which the kinetics in blood was evaluated via direct sampling, while the kinetics in kidney, liver, and brain was assessed via serial near-infrared imaging in vivo. The authors designed a five-compartment pharmacokinetic model that satisfactorily fits the data for single VHHs, VHH trimers, and iron oxide nanoparticles conjugated to VHH trimers. The methods used proved to be feasible and are a promising approach to future study of the pharmacokinetics of candidate molecular contrast agents for magnetic resonance imaging.

Magnetic iron oxide nanoparticles are potential contrast agents for magnetic resonance imaging and magnetic particle imaging. Baki and co-workers [5] modified single-core iron oxide nanoparticles via albumin coating and assessed their colloidal stability under different sodium chloride concentrations using transmission electron microscopy and differential centrifugal sedimentation; the successful surface modification and the optimal colloidal stability were confirmed by magnetic particle spectroscopy and nuclear magnetic resonance measurements. The colloidal stability and the preserved magnetic characteristics in a physiological environment make these tailored nanoparticles suitable as molecular contrast agents for magnetic resonance imaging and magnetic particle imaging.

Woźniak and co-workers [6] reviewed different techniques (optical, ultrasound, magnetic resonance and nuclear imaging, and computed tomography) applied to theranostics nanoparticles. The authors underline how these techniques enable elucidation of the molecular interactions of these nanoparticles in different diseases, improving the process of diagnosis and therapy in personalized medicine. 

Dopamine-related tracers are a powerful tool used to assess the status of presynaptic nigrostriatal terminals in the brain; Palermo and co-workers [7] illustrate the use of positron emission tomography and single-photon emission tomography imaging with specific dopamine-related tracers as an indirect imaging biomarker of Parkinson’s disease, and carefully discuss the actual diagnostic and prognostic significance of this imaging approach.

Aptamers are short, single-stranded, non-coding DNA or RNA nucleotides that specifically bind molecular targets and may be functionalized via radioisotope labeling for use as diagnostic and therapeutic agents. Due to their high specificity and adjustable binding affinities, aptamers targeting HER2 are promising agents in nuclear medicine for the early detection, diagnosis and potential treatment of HER2-positive breast cancer. In their review, Vi and co-workers [8] describe several HER2 aptamers and discuss their possible translation into clinical practice as novel primary treatments, or as adjuvant therapies for HER2-positive breast cancer.

After describing the concepts and characteristics of general optical coherence tomography systems, Wang and co-workers [9] describe the recent progress in the application of contrast agents, focusing on the combination of optical coherence tomography and nanoparticle contrast agents in the nanomedical field. The authors discuss the benefits and limitations of this imaging technique, for which a wide range of nanomedical applications is predicted, as they combine sensitive molecular information with high-resolution morphological images.

Among the existing contrast agents for magnetic resonance imaging, smart nanoprobes that respond to selective stimuli (e.g., specific enzyme activities or pH) may provide information on tissue and cell functional features. This subject was reviewed by Wang and co-workers [10], who focused on the design and application of environment-responsive nanoprobes that have recently been developed to specifically detect cell populations, based on their different tissue milieu. Using these nanoprobes, the contrast between diseased and normal tissues may be successfully improved.

Elucidating the interactions between the nanoconstructs and the biological environment is crucial in nanomedicine, and transmission electron microscopy provides direct evidence of the uptake and biodistribution of nanoparticles, as well as of their relationships with the cell and tissue components. My paper [11] presents an overview of the transmission electron microscopy techniques suitable for exploring the effects of nanoconstructs on biological systems, and demonstrates the informational value of ultrastructural morphology, histochemistry and microanalysis in nanomedical research.

Overall, this Special Issue confirms the key contribution of molecular imaging techniques, which support and foster the creativity and inventiveness of scientists engaged in nanomedical research.
